# Green Communication in Internet of Things: A Hybrid Bio-Inspired Intelligent Approach

**DOI:** 10.3390/s22103910

**Published:** 2022-05-21

**Authors:** Manoj Kumar, Sushil Kumar, Pankaj Kumar Kashyap, Geetika Aggarwal, Rajkumar Singh Rathore, Omprakash Kaiwartya, Jaime Lloret

**Affiliations:** 1 School of Computer and Systems Sciences, Jawaharlal Nehru University, New Delhi 110067, India; manoj26_scs@jnu.ac.in (M.K.); skdohare@mail.jnu.ac.in (S.K.); pankaj76_scs@jnu.ac.in (P.K.K.); 2 School of Science and Technology, Nottingham Trent University, Nottingham NG11 8NS, UK; geetika.aggarwal@ntu.ac.uk; 3 Department of Computer Science, Cardiff School of Technologies, Cardiff Metropolitan University, Cardiff CF5 2YB, UK; rsrathore@cardiffmet.ac.uk; 4 Department of Communications, Universitat Politècnica de València, 46022 Valencia, Spain; jlloret@dcom.upv.es; 5 School of Computing and Digital Technologies, Staffordshire University, Stoke ST4 2DE, UK

**Keywords:** Internet of Things, chicken swarm optimization, genetic algorithm, energy optimization

## Abstract

Clustering is a promising technique for optimizing energy consumption in sensor-enabled Internet of Things (IoT) networks. Uneven distribution of cluster heads (CHs) across the network, repeatedly choosing the same IoT nodes as CHs and identifying cluster heads in the communication range of other CHs are the major problems leading to higher energy consumption in IoT networks. In this paper, using fuzzy logic, bio-inspired chicken swarm optimization (CSO) and a genetic algorithm, an optimal cluster formation is presented as a Hybrid Intelligent Optimization Algorithm (HIOA) to minimize overall energy consumption in an IoT network. In HIOA, the key idea for formation of IoT nodes as clusters depends on finding chromosomes having a minimum value fitness function with relevant network parameters. The fitness function includes minimization of inter- and intra-cluster distance to reduce the interface and minimum energy consumption over communication per round. The hierarchical order classification of CSO utilizes the crossover and mutation operation of the genetic approach to increase the population diversity that ultimately solves the uneven distribution of CHs and turnout to be balanced network load. The proposed HIOA algorithm is simulated over MATLAB2019A and its performance over CSO parameters is analyzed, and it is found that the best fitness value of the proposed algorithm HIOA is obtained though setting up the parameters$\;pop_{size}=60$, number of rooster$\;N_{r}=0.3$, number of hen’s $N_{h}=0.6$ and swarm updating frequency θ=10. Further, comparative results proved that HIOA is more effective than traditional bio-inspired algorithms in terms of node death percentage, average residual energy and network lifetime by 12%, 19% and 23%.

## 1. Introduction

Over the years, revolutionary development in IoT devices has opened the paradigm for dynamic sensing technology that provides seamless communication over the Internet [1]. Wireless sensor networks (WSNs) are prominently used for the collection of data and communication over the fifth generation (5G) and beyond 5G IoT network envision as sixth generation (6G) technology [2]. Moreover, the combination of IoT networks with WSNs has many potentials in various applications, such as precision agriculture, intelligent transport systems, health care, smart cities, military, environment and habitat monitoring, environment anomalies and human intrusion detection [3,4]. However, with all these remarkable properties, the fallout in the unbalanced energy consumption and lower lifetime of battery-enabled IoT devices limits the seamless communication of intelligent devices over the IoT network. Therefore, energy-efficient communication over 5G and beyond 5G (6G) enabled IoT devices is the utmost concern in IoT network use cases.

Clustering is a robust and scalable approach to lower energy consumption with better network throughput [4,5]. It is widely studied as probability-based [6,7,8,9], weight-based [10,11,12] and heuristic-based approaches [13,14,15,16,17,18] for conservation of network energy. Moreover, local decision and uncertainties of the network dynamics have a huge impact on energy consumption in the optimal cluster head (CH) selection as designated data forwarder. Further, the problem of CH selection is non-deterministic polynomial hard (NP) since optimal data aggregation cannot be efficiently solved in polynomial time to ensure balanced energy consumption in each round using a probability- and weight-based approach [19]. Recent studies have shown that meta-heuristics approaches are more suitable for approximately solving NP problems for CH selection [20]. Consequently, proper optimization methods such as fuzzy logic inference [13,14], bat algorithm [15], particle swarm optimization [16,17,18], differential evolutionary and harmony search [19,20], genetic algorithm (GA) [21], and bio-inspired chicken swarm optimization (CSO) have the potential to be effectively used for finding the optimal number of CHs [22,23,24].

A critical investigation of CSO techniques concluded that it has better ability for IoT network-centric feature selection with faster convergence rate over fuzzy logic and genetic algorithm due to the effective balance between network uncertainty and finding the parameter optima [17]. In fuzzy logic, the output depends upon only the knowledge base rule, which is robust in nature, but fuzzy logic may not be applicable in frequent network environment change scenarios, whereas GA has the ability to adapt the environment precisely and CSO has better hierarchal classification and speed reduction design in size for optimization problems with maximum accuracy [25]. Therefore, in this paper, integrating the above three features of the mentioned technique, we propose a novel Hybrid Intelligent Optimization Algorithm (HIOA) to optimize the overall energy consumption in the network by rotating the role of CHs. The presented HIOA integrates Fuzzy logic (FL) and chicken-swarm genetic optimization (CSGO) algorithms that inherently address the problem of repeatedly choosing the same IoT nodes as CH during transmission rounds. CSGO simulates foraging activity by dividing the chicken into smaller groups. In each group, every chicken moves toward the optimal one simultaneously, which motivates the idea of rotating CH in each time slot. The major contributions of the proposed model are as follows:

- The system model includes cluster-based IoT architecture with aid the feature of cloud network, where energy consumption of the cluster network is evaluated.
- An energy optimization problem is formulated in terms of a fitness function that minimizes the intra- and inter-cluster distance in the IoT network.
- We present HIOA to generate an optimal set of CHs to minimize the overall energy consumption. This employs FL for the creation of the initial population. Further, CSGO divided the IoT node into a hierarchal structure to increase the population diversity that optimizes the formulated fitness function using crossover and mutation.
- Finally, extensive simulation over different CSGO parameters and comparison with the state-of-the-art algorithms has been performed for critical performance evaluation.

The rest of the paper is divided into the following sections. Section 2 shows the related literature. Section 3 describes the presented system model, problem formulation and proposed algorithm in detail. In Section 4, the simulation results and analysis are discussed. The conclusion of the work and future perspective are presented in Section 5.

## 2. Related Works

As mentioned above, recent studies [13,14,15,16,17,21,22,23,24] through simulation have observed that artificial intelligence and precisely bio-inspired techniques are preferable to traditional probabilistic and deterministic approaches subject to optimizing the energy consumption in IoT networks. In [17,21], the authors proved the proposed algorithm based on CSO obtains excellent performance over traditional approaches such as PSO, FL and GA for robust beam-forming approach, whereas authors in papers [24,25] CSO-based clustering routing protocol plays a significant role in reducing energy consumption over integration with bio-inspired approaches.

In paper [26], a Fuzzy-based routing protocol (FRP-LEACH) is proposed to enhance the traditional Low Energy Adaptive Clustering Hierarchy (LEACH) protocol that forms the clusters in an energy efficient manner. The proposed FRP-LEACH works over cross-layers, and the authors claim that cloud-based services such as the proposed algorithm protect medical staff and patients from the ongoing COVID-19 pandemic. However, the proposed algorithm is restricted to a lower dataset as the COVID-19 dataset increases, then fuzzy logic fails to adapt the changes delay in transferring the packet and node death ratio increases according to data growth rate. The authors in paper [27] enhanced the performance of LEACH using a fuzzy logic inference system and renamed LEACH-FL. However, both approaches carry forward the clustering process efficiently by considering the fuzzy logic inputs as closeness to the base station and residual energy but left out the node density parameter. This cause’s optimum number of CHs generated through approaches to cover the entire network was not ensured. 

In [28], authors have proposed EC-PSO models based on a standard PSO approach to optimize the energy consumption of the network to overcome the hotspot problem. When some of the sensor nodes may be left out from the coverage area of CHs and live spontaneously, they are called isolated nodes. These nodes continuously search for CHs and forces to communicate base station directly to exhaust more energy. However, the authors completely ignore the node distance from the base station, which causes it to affect the fitness function tremendously and ultimately consume uneven energy in each time slot. In [29], the authors proposed an enhanced version of a cluster-based genetic algorithm referred to as CRCGA by coding the fitness function to minimize the energy consumption and load balancing of the network. This proposed algorithm mainly considers three factors: formation of clusters, finding the best route and then maintaining the clusters appropriately in each time slot. Further, adaptive round-trip time is considered over the traditional TDMA schedule to further improve network performance. However, CRCGA outperforms the traditional algorithm but fails to consider the scenarios of different sink node positions. In the paper [30], authors have reduced the localization error in WSN using the CSO technique, and further statistical analysis compared to benchmarks optimization technique PSO and GA reveals that CSO provides an upper hand to robustness, precision and performance in terms of convergence speed over the IoT network. To boost the performance of CSO, a cuckoo search is integrated called CSCSO [31] to find the optimal route for data transfer between node and base station. An enhanced version of LEACH using CSO is presented as LEACH-PSO [32] to form optimal clusters and routing paths. Whereas in the paper [33], authors have addressed the problem of balance between power supply and demand using the CSO technique in the residential area and tertiary industry. Further simulation proved that the improved CSO algorithm outperforms in interruptible load scheduling over peak demand in real time over the GA and PSO algorithms. The above-mentioned algorithms missed the multi-hop routing scenario that extends to inter-clustering routing and intra-clustering routing exchange a greater number of packets that maximize the overall energy consumption of the network. 

From the above comparative study of Table 1, it is clearly demanding that bio-inspired CSO and genetic candidate are the best suited approach to handle the uneven clusters formation and efficiently optimize the overall energy consumption. In this paper, clustering is divided into two phases. (i) Fuzzy logic is used to produce tentative CHs. (ii) Thereafter, CSO with GA is used to produce the final optimal number of CHs at each round considering the minimization of total energy consumption per round focuses on enhancing the IoT network lifetime.

## 3. Green Communication in the Internet of Things: A Hybrid Bio-Inspired Intelligent Approach

### 3.1. Network Model

In the energy-constraint IoT network, all the smart IoT nodes are deployed in the environment randomly. These sensor nodes form clusters as a Fuzzy logic-based chicken swarm genetic optimization (HIOA) algorithm. Only CH is able to send aggregated data to nearby edge nodes. Finally, edge node transferred data to the cloud network for data storage that can be used for data analytics with the following assumptions about IoT nodes below. All the smart IoT sensor nodes are homogenous in nature in terms of energy and computation capabilities (such as data collection, transmission and data aggregation). All these wireless IoT nodes periodically observe the environment and send data to their nearby CH. Initially, all the IoT nodes start with an equal quantity of energy stored in the battery. Using the received signal strength indicator (RSSI), IoT nodes calculate their distance from edge nodes by finding their co-ordinate ($X_{l},Y_{l}$). An equal amount of energy is consumed by the symmetrical channel in packet transfer in either direction from A to B or B to A. In addition, IoT nodes can adjust their transmission power for packet transfer according to distance either from the edge node or CH. The operational period is divided into two phases: the setup phase and the steady state phase. A crystal-clear observation of a single transmission period is further explained in Section 3.3.4 Transmission Procedure of HIOA Algorithm.

### 3.2. Energy Model

The IoT nodes set their states between transmitter and receiver before the start of the transmission period. The total energy ($E_{S}\left(y,z\right)$) consumed to send y bit of packet is twofold (a) energy $\left(E_{T}\right)$ exhaust in transceiver circuit to process the packet (b) energy $\left(E_{A}\left(y,z\right)\right)$ consumption takes place to amplify the y bit of packet over z distance either in free-space energy ($\partial_{fs}$) or multipath energy ($\partial_{mp}$) corresponds to threshold distance $z_{0}$ similar to the first order radio model [3] as follows:

(1) $$E_{S}\left(y,z\right)=E_{T}+E_{A}\;\left(y,z\right)=\{\begin{array}{c} y\;E_{T}+y\;\partial_{fs}\;z^{2},\;if\;z<z_{0}\;\\ \;y\;E_{T}+y\;\partial_{mp}\;z^{4},\;if\;z\geq \;z_{0}\; \end{array}$$

Additionally, energy ($E_{r}$) consumed by IoT node to receive y bit of packet as follows:

(2) $$E_{R}\left(y,z\right)=yE_{T}$$

We consider N IoT nodes are evenly distributed into W clusters such that N/W nodes are allocated to each cluster. Further, CHs use the TDMA slotted period to get data from their member IoT nodes, where IoT nodes are able to transmit the sensed data to their CH only in wakeup mode after that switch to sleep mode. The total energy ($E_{P}$) exhaust by each CH in a single transmission period is the energy payoff in (a) ($E_{g}$) gathering the data from its member’s node and (b) ($E_{e}$) transmitted the received data to the edge node as aggregated data following the multipass model can be written as:

(3) $$E_{P}=E_{g}+E_{e}$$

where, $E_{g}=yE_{T}\left(\frac{N}{w}-1\right)$, and $E_{e}=yE_{ay}\frac{N}{W}+yE_{T}+y\partial_{mp}\;z_{pe}^{4}$ is consumed energy in the multipass model. $E_{ay}\;$represents the data aggregation energy and $y_{ze}$ denote the distance between CH and edge node. The energy consumed ($E_{c}$) by child IoT nodes to transfer gathered data to their parent CH uses a free space model over average distance $(z_{cp}=\left(1/2\pi \right)\left(R^{2}/w\right)$, *R* is diameter of network) between parent and child node in one round as follows:

(4) $$E_{c}=yE_{T}+y\partial_{fs}z_{cp}^{2}$$

Overall, each cluster exhausts the total energy is the summation of energy consumed by parent CH and its child IoT nodes during one round as follows:

(5) $$E_{cluster}=E_{P}+E_{c}=y\left(2\;N\;E_{T}+NE_{ay}+w\;\partial_{mp}\;z_{pe}^{4}+w\;\partial_{fs}z_{cp}^{2}\right)$$

(6) $$=y\left(2\;N\;E_{T}+NE_{ay}+w\;\partial_{mp}\;z_{pe}^{4}+w\;\partial_{fs}\;\frac{1}{2\pi }\;\frac{R^{2}}{w}\right)$$

### 3.3. Hybrid Intelligent Energy Optimization

**Figure 1:**
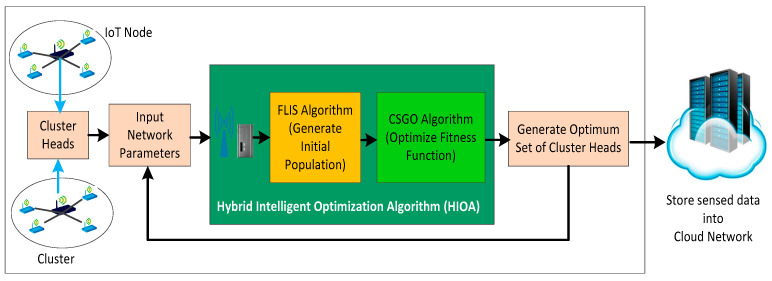
Block diagram of HIOA.

The primary objective of the proposed HIOA approach is to generate an optimal set of CHs to minimize the overall energy consumption and prolong IoT network lifetime, as shown in Figure 1. It consists of two optimization phases. First, a tentative set of CHs is selected through fuzzy logic inference system (FLIS) optimization. In the second optimization phase, the chicken swarm genetic optimization (CSGO) algorithm uses the output of the FLIS labeled as input and treated as the first initial population. Further, the CSGO algorithm optimizes the election process by choosing the appropriate IoT node as CH to successfully bring out optimum clustering operation.

#### 3.3.1. Adapted Fuzzy Logic Interference System for Green Communication

In the first optimization phase, a tentative number of CHs is selected based on three parameters in an IoT network: residual energy ($I_{re}$), node density ($I_{nd}$), distance to edge node ($I_{de}$). The reason behind the selection of these parameters as follow: $I_{re}$ relate to IoT node becoming a CH having enough energy to successfully execute the operation of clustering. $I_{nd}$ relates to the number of neighboring IoT nodes corresponding to the selected node as CH, if $I_{nd}$ is in sparse of nature to the CH, then communication cost increases. Moreover, the worst-case cluster member’s nodes exhaust all energy in the transmission of observed information because it is far from CH. $I_{de}$ defining the CH should be at an optimal position so that it manages the communication cost both from cluster member’s nodes and edge nodes.

**Figure 2:**
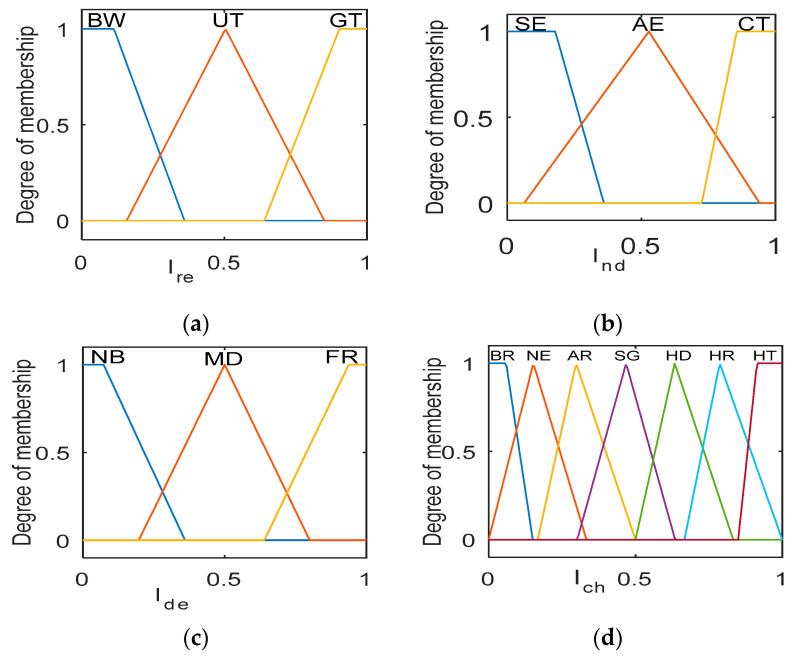
Membership function: (**a**) Residual energy (***I_re_***), (**b**) Node density (***I_nd_***), (**c**) Distance to edge node (***I_de_***), and (**d**) Probability chance (***I_ch_***).

The linguistic variable for mamdani type FLIS as input: $I_{re}$ = {below (BW), upright (UT), great (GT)}, $I_{nd}$ = {sparse (SE), average (AE), compact (CT)} and $I_{de}$ = {nearby (NB), midway (MD), far (FR)}. These parameters are fed into FLIS and in turn output probability as a chance calculated for an IoT node to become CH or not. The linguistic variable of the output probability chance as follow: $I_{ch}=${beginner (BR), naive (NE), amateur (AR), strong (SG), hard (HD), harder (HR), hardest (HT)}. The extreme linguistic variables are denoted by trapezoidal membership functions (MFs), and the middle ones are from triangular MFs, as shown in Figure 2a–d. The If-Then rules (input$\;3^{3}=27$) for evaluation of output probability are presented in Table 2.

The FLIS evaluates the output probability in four steps as follows: (i) Fuzzification—this step creates MFs of the crisp input variables according to intersection point. (ii) Fuzzy rule-base—all 27 If-Then rules executed parallel on the given three input variables to generate single output. This can be done by using a fuzzy minimum AND operator from the selection of three input MF parameters. (iii) Aggregation—as there is multiple output value generated corresponding to 27 rules, to aggregate as one single output, maximum union operator OR is used. (iv) Defuzzification—the center of area is used for defuzzification of aggregated value into a crisp output. After the selection of CHs process, the remaining nodes join the nearest CHs through the join message.

#### 3.3.2. Adapted Chicken Swarm Genetic Optimization for Green Communication

**Figure 3:**
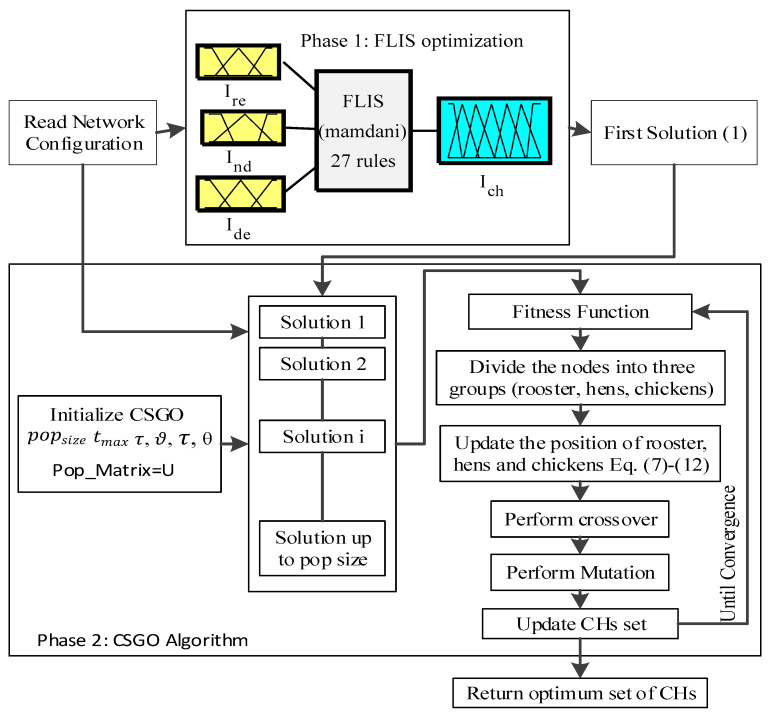
Workflow of the HIOA Algorithm.

In the second optimization phase, the set of tentative CHs resulting from FLIS optimization serves as the initial population of the CSGO to generate a better set of CH compared to the first FLIS optimization phase. In the proposed CSGO algorithm, binary representation is used to represent the IoT nodes as CH (1) or a normal IoT node (0). The binary index ($b_{IN}$) value of an IoT node can be calculated using the sigmoid function as follows:

(7) $$b_{IN}=\{\begin{array}{c} 1,\;if\;sigm\;f\left(I_{ch}\right)>0.5\\ 0,\;otherwise \end{array}$$

where, $sigm\;f\left(I_{ch}\right)=1/1+e^{-I_{ch}}$ represent the sigmoid function. The CSGO includes adapting GA crossover and mutation processes into traditional chicken swarm optimization to enhance the diversity in the population. In the presented algorithm, each cluster is represented as pack classified into Rooster, hens and chicks. Chickens have the best fitness value represented as Rooster (CHs) and chicks are the chickens with the worst fitness value. Most chickens are hens labeled as normal IoT nodes. The hens and chicks form mother–child relationships arbitrarily. Moreover, the mother–child relationship and dominance relation are unaltered in a pack and updated every distinct (*θ*) swarm updating frequency as time steps. The position of the rooster is updated using $rand\left(0,\sigma^{2}\right)$ Gaussian distribution with mean zero and standard deviation $\left(\sigma^{2}\right)$ as follow:

(8) $$Y_{m,D}^{t+1}=Y_{m,D}^{t}*\left(1+rand\left(0,\sigma^{2}\right)\right)$$

(9) $$\sigma^{2}=\{\begin{array}{c} 1\;if\;𝒻\left(F_{m}\right)\leq 𝒻\left(F_{r}\right)\\ \exp\left(\frac{𝒻\left(F_{r}\right)-𝒻\left(F_{m}\right)}{\left|𝒻\left(F_{m}\right)\right|+£}\right),\;otherwise \end{array},\;r,m\;\in \left[1,N\right],\;r\neq m$$

where, $Y_{m,D\;}^{t+1}$ represent the position of *m*th rooster in the D-dimension space, $𝒻\left(F_{r}\right)$ define the fitness function of the randomly selected *r*th rooster and £ is the smallest constant to avoid zero-error division. The position of the *m*th hens’ is updated as follows:

(10) $$Y_{m,D}^{t+1}=Y_{m,D}^{t}+\rho_{1}*rand\left(Y_{\omega 1,D}^{t}-Y_{m,D}^{t}\right)+\rho_{2}*rand\left(Y_{\omega 2,D}^{t}-Y_{m,D}^{t}\right)$$

(11) $$\rho_{1}=exp\left(\frac{𝒻\left(F_{m}\right)-𝒻\left(F_{\omega 1}\right)}{\left|𝒻\left(F_{m}\right)\right|+£}\right)\;\mathrm{and}\;\rho_{1}=\exp\left(𝒻\left(F_{\omega 2}\right)-𝒻\left(F_{m}\right)\right)$$

where, $\rho_{1}$, $\rho_{2}\in \left[1,2\ldots .N\right]$ are the index of randomly chosen rooster and chickens (hens’ or chicks), respectively, such that $\rho_{1}\neq \rho_{2}$. The rand( ) function generates a uniform random number between 0 and 1. Similarly, the position of *m*th chicks follows their mother position (FL) as follows:

(12) $$Y_{m,D}^{t+1}=Y_{m,D}^{t}+FL\left(Y_{M,D}^{t}-Y_{m,D}^{t}\right)$$

where, $Y_{M,D}^{t}$ stands for the position of *m*th chick’s mother, such that M∈[1,n]. FL( ) function allows chick’s to choose any random value between zero and two. The proposed HIOA algorithm (refer to Algorithm 1) for the generation of the best set of CH works in two phases shown in Figure 3. The first phase generates the tentative list of CHs using FLIS. The second phase (CSGO) generates the optimum set of (rooster) CHs and genetic operators such as crossover and mutation are applied over hens’ and chicks to enhance their fitness value. In addition, CSGO includes three steps: (i) initialization step—set the K number of CHs obtained from FLIS optimization, number of rooster ($N_{r}$), hens$\;(N_{h}$) and chicks $(N_{c}$), population size ($pop_{size}$), maximum number of swarm updating frequency (θ), single point crossover (ϑ) and mutation rate (τ) with maximum iteration. (ii) Selection phase—it updates the position of rooster, hens’ and chicks using Equations (7)–(12). Moreover, crossover and mutation operators were applied over hens’ and chicks to enhance their fitness value. (iii) Output step—individuals having the best fitness value generated as the optimum set of CHs.
**Algorithm 1:** HIOA**Begin**Read network configuration***First phase:*FLIS optimization*****Input:*** $I_{re}$, $I_{nd}$, $I_{de}$Execute the FLIS engine based on rules define in Table 2Return the chance of an IoT node selected as CH***Output:*** Tentative set W number of CH***Second phase:*****CSGO algorithm*****Initialization:*****a**.assign $pop_{size}$, $t_{max}$= maximum generation, τ, ϑ, τ, θ**b**.Initialize the population matrix U by random values from 0 and 1.**c**.Include the output of FLIS (set of k number of CHs) as one feasible solution.***Selection:***Reconstruction of infeasible solution takes places those have CHs less than k.Calculate the fitness value of each row of U.Optimum set ($O_{CH}$) = row of U having best fitness value.**For** t = 1 to $t_{max}$ **do****If** (t mod θ==0 || t==1) **then****a**.Sort the individuals into ascending order according to their fitness value.**b**.Divide into three category rooster ($N_{r}$), hens’$\left(N_{h}\right)$ and chicks$\;(N_{c})$.**c**.Determine the relationship between mother-child in a pack.**End If****for** each $m^{th}\;$individuals in each Y row of U **do** // $pop_{size}$ **If m== rooster then**   update its position using Equation (8). **Else if m== hen then**   update its position using Equation (10). **Else if m==chick then**   update its position using Equation (12). **end if** Convert Y into binary form using Equation (7) // Single-point Crossover **for** $D=N_{r}+1$ to $pop_{size}$ **do** generate two child offspring $y_{o1}\;\mathrm{and}\;\;y_{o1}$ from two parent chromosome $Y_{D\;}$and $Y_{D+1}$ set $Y_{D\;}=y_{o1}$ and $Y_{D+1\;}=y_{o2}$ **end for**   // end of for loop in line no. 22 // Mutation **for** $D=N_{r}+1$ to $pop_{size}$ **do**  r= generate rand(0,1)  **if** r<τ **then**   **integer** $\;\rho_{1}=$ generate rand(0,n)   **if** $Y\left(\rho_{1}\right)==1$ **then**   set $Y\left(\rho_{1}\right)=0$  **else**   set $Y\left(\rho_{1}\right)=1$   **end if**   **end for**  // end of for loop in line no. 27  Reconstruction of infeasible solution takes places those have CHs less than k.  Updating the fitness value of each Y row of U.  If fitness value of new solution is better than previous one, then update optimum CH set with new solution. **End for** // end of for loop in line no. 17.**End for** // end of for loop in line no. 14**Output:** Return the optimum set $\left(O_{CH}\right)\;$ of CHs.

#### 3.3.3. Complexity Analysis of HIOA

The presented HIOA algorithm works in two phases. The time complexity of FLIS optimization consists of maximum comparison for each elected CH is ($N^{2}-N$), where N is the number of IoT nodes. Thus, the time complexity of the first phase is$\;O\left(N^{2}\right)$. In the CSGO phase, the initialization step takes a constant time of O(1). In addition, line no. 11 to line no. 13 are executed in O(1) time. Further, the computational complexity of the second phase mainly depends upon line no. 14 up to line 43, which consists of four nested loops. Line no. 14, 17, runs up to maximum generation$\;t_{max}$ and possible solution$\;pop_{size}$, respectively; further Line no. 24 and 29 both executed up to N number of nodes. Therefore, the time complexity of the second phase can be evaluated as$\;O\left(1\right)+O\left(1\right)+O\left(t_{max}\times pop_{size}\left(N+N\right)\right)$ ≈ O(N), where $t_{max}$ and $pop_{size}$ are very small compared to the number of IoT nodes and can be neglected. Thus, the overall time complexity of the presented HIOA algorithm is the summation of first phase and second phase$\;\left[O\left(N^{2}\right)+O\left(N\right)\right]$.

#### 3.3.4. Operational Procedure for HIOA

An operational period for the presented HIOA algorithm operates in a setup and steady-state period that is repeated in each round. Thus, each IoT node has the chance to play the role of CHs in blanching the energy consumption in the IoT network. Firstly, in the setup period, CH election and binding of IoT nodes subject to cluster formation takes place. To get knowledge about IoT nodes, edge nodes advertise beacon messages over the network. In turn, IoT nodes respond with their ID, co-ordinate and residual energy. Using the RSSI model, IoT nodes calculate their distance from the edge node by finding their co-ordinate ($X_{l},Y_{l}$) as follow:

(13) $$\mathrm{RSSI}=-\left(10\varphi \;log_{10}\;z_{ie}+A\right)$$

where, φ represents the coefficient of signal propagation parameter alias exponent, $z_{ie}$ is the usual distance between edge node to an IoT node and A denotes the obtained signal strength in one meter distance without obstacle. Further, the edge node uses the presented HIOA for the election of K number of CHs. Furthermore, edge nodes send messages to each IoT node in the K pack to inform nodes as CHs. Each IoT node in the pack advertises its role as cluster member CM or CHs containing its ID. Those remaining nodes that do not belong to any pack choose the nearby CH based on minimum communication cost through the join message. Second, in the steady state period, the major goal is to avoid data collisions occurring during the gathering of data by parent CH from its child CM nodes. Then, CHs schedule the data gathering using the TDMA technique. Now, CM sends their observed data to their respective CH that include ID and residual energy. This local information is useful in deciding on the role of CH for the next round in a pack. CH applies a data aggregation algorithm to remove redundancy in the gathered data. Finally, CH sends a packet of fixed size to the edge node with the local information of CM’s.

## 4. Results and Discussion

The performance of the HIOA algorithm is compared with a state-of-the-art algorithm using MATLAB 2019A. This section is divided into two parts: (i) CSGO parameter analysis and (ii) network parameter analysis. In addition, 200 IoT nodes are randomly distributed over the IoT network size 150 × 150 m^2^ with edge node set at the corner position of the network. The initial power of the nodes is 1 J, similar to the first order radio model used in paper [20]. CSO is initializing with a random solution in the search space and applied to minimize the fitness function that yields a single solution with all features selected as the initial solution. For CSO, the maximum generation is set to be 60, the number of roosters is between 0.1 to 0.4 percentages randomly, and the number of hens is between 0.2 to 0.8 randomly. The list of other parameters and their values is listed in Table 3, which is similar to paper [29,31].

### 4.1. CSGO Parameter Analysis

In this subsection, the consequences of four different parameters on the fitness performance of the presented algorithm HIOA are analyzed. These parameters are (i) population size ($pop_{size}$) (ii) number of rooster ($N_{r}$) (iii) number of hens’ ($N_{h}$) and (iv) swarm updating frequency θ. In addition, the number of chicks can be evaluated as $N_{c}$ =$\;N-N_{r}-N_{h}$.

**Figure 4:**
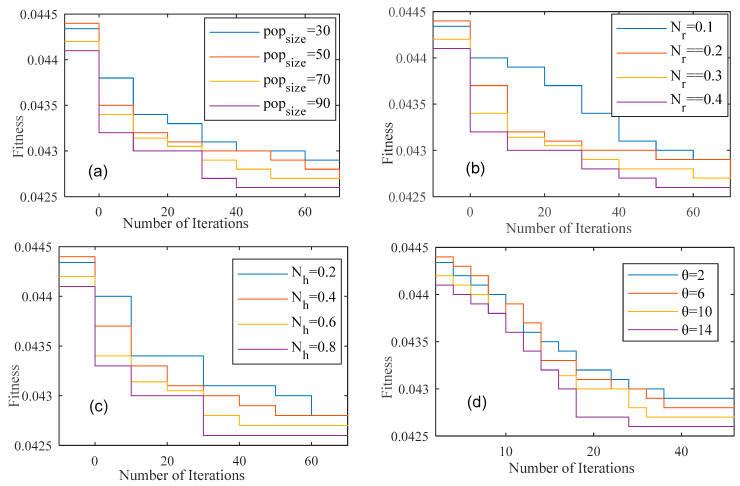
Optimization performance of HIOA over iterations (**a**) *pop_size_* (**b**) *N_r_* (**c**) *N_h_* (**d**) *θ*.

From the above analysis of the result from Figure 4a–d, we observed that the presented algorithm achieves a better fitness value of about 0.625 within 60 iterations and after that more iteration, the variation in fitness value is negligible. This can be attributed to the reason the presented algorithm evaluates the fitness value on the basis of three optimization functions: inter-cluster, intra-cluster distance and energy consumption. This reveals that the presented algorithm is able to balance the energy consumption and average distance properly. Thus, the best fitness value of the proposed algorithm HIOA is obtained through setting up the parameters $pop_{size}=60$,$\;N_{r}=0.3$, $N_{h}=0.6$ and θ=10. In addition, the number of chicks can be evaluated as $N_{c}=1-0.3-0.6=0.1$. In the next upcoming simulation, the above parameters are set with their values.

### 4.2. Network Parameter Analysis

In this section, a comparison of the HIOA with LEACH-FL [27], CRGA [29] and EC-PSO [28] algorithms has been analyzed to show the effectiveness in terms of network lifetime, average energy consumed per node at each round and the total average energy consumed per round.

#### 4.2.1. Comparison of Active Nodes over Rounds

**Figure 5:**
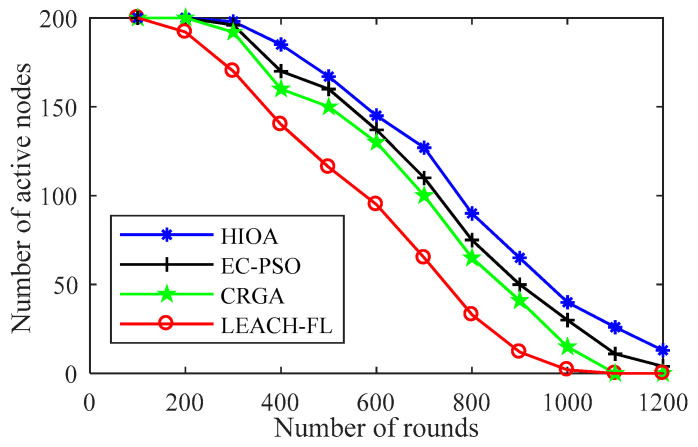
Number of active nodes over round.

In Figure 5, the number of active IoT nodes is plotted per round to show the effectiveness of the proposed algorithms compared to state-of-the-art algorithms. It is evident from the result for the starting phase of all the algorithms, up to 300 rounds around 98% IoT nodes are alive/active except in LEACH-FL only 87%. Further, on enhancement in rounds, the proposed algorithm shows the best performance compared to EC-PSO, CRGA and LEACH-FL. This is due to the fact that each chicken in the proposed algorithm acts as an agent, which updates its position in the network areas to become CH or not. In addition, this agent (chicken) has information about the whole group (cluster) and fairly balances the energy consumption load using GA crossover and mutation operator. Further, the EC-PSO algorithm does not have the ability to multi-swarm optimization and lacks adaptation to changes in the network. Whereas, CRGA do not has agent so that keep information’s of other chromosomes in a population. It is also worth noting that the number of active nodes in LEACH-FL is only 20 after crossing 800 rounds and further increments in the number of rounds in turn all the nodes die. This result confirms that LEACH-FL is not able to achieve fairness in network load balance and residual energy of IoT nodes. Thus, overall, the proposed algorithm HIOA outperforms EC-PSO, CRGA and LEACH-FL by 12%, 19% and 29%, respectively, in terms of the number of active nodes remaining in the 900 rounds.

#### 4.2.2. Comparison of Network Lifetime over Rounds

**Figure 6:**
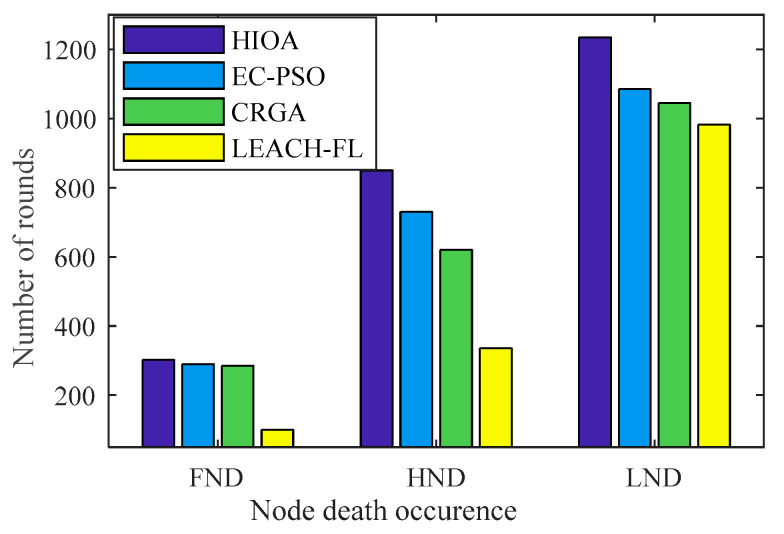
Network lifetime over round.

A comparison of the network lifetime of the proposed algorithm and state-of-the-art algorithms is shown in Figure 6. The network lifetime is the time at which the first node dies (FND) or half of the nodes die (HND) and the last node dies (LND). It can be clearly observed from the result the proposed algorithm HIOA achieves better lifetime in terms of FND is 11%, 18% and 48% from EC-PSO, CRGA and LEACH-FL, respectively. Further, HND is 27%, 35% and 58% compared with EC-PSO, CRGA and LEACH-FL, respectively. Furthermore, in terms of LND is 35%, 46% and 81% compared to EC-PSO, CRGA and LEACH-FL, respectively. This can be attributed to the reason that the proposed algorithm uses both fuzzy logic optimization with a chicken swarm genetic algorithm that helps in building a more feasible solution rather than only using particle swarm optimization in EC-PSO or a genetic algorithm in CRGCA. This is also observed from the result that LEACH-FL shows the worst performance in terms of lifetime, where FND, HND and LND occur at 100, 336 and 982 rounds. It is due to the reason LEACH-FL selects the CH based only on residual energy and node distance, where node density is left out. There is no optimization, such as PSO, or GA is used to enhance performance.

#### 4.2.3. Comparison of Average Energy Consumed over Rounds

**Figure 7:**
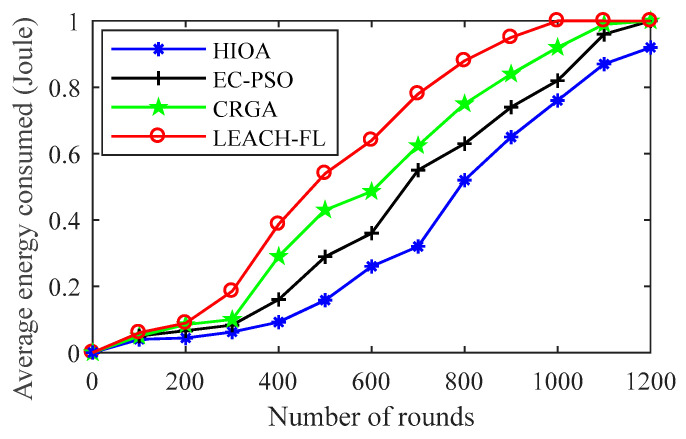
Average energy consumed over round.

A comparison of average consumed energy over rounds of the proposed algorithm HIOA and state-of-the-art algorithms is shown in Figure 7. Initially, all the state-of-the-art algorithms except LEACH-FL [27], up to 300 rounds, consume almost similar amounts of energy. Thereafter, on the enhancement of the number of rounds, the proposed algorithm HIOA consumes much less energy than other state-of-the-art algorithms and goes up to above 1200 rounds with a consumption of 0.75 Joule. In addition, it can be also observed that CRGA [29] and EC-PSO [28] consume almost 0.95 Joule and 0.85 Joule of energy within 972 rounds and 1030 rounds, respectively. This is due to the fact presented HIOA algorithm inherit CSGO that designated to inmate multi-swarm optimization group with crossover and mutation technique of genetic algorithm, which enhances the efficient use of energy in the IoT network. EC-PSO is a single group swarm optimization technique that is lacking in the generation of feasible solutions of CHs throughout the IoT network. In addition, the fitness function of CRGA only includes residual energy parameter and left the inter-cluster distance and intra-cluster distance parameter, so that selection of CHs is not optimal. Thus, overall, the proposed algorithm HIOA outperforms EC-PSO, CRGA and LEACH-FL by 17%, 26% and 38%, respectively.

#### 4.2.4. Comparison of Average Residual Energy over Rounds

**Figure 8:**
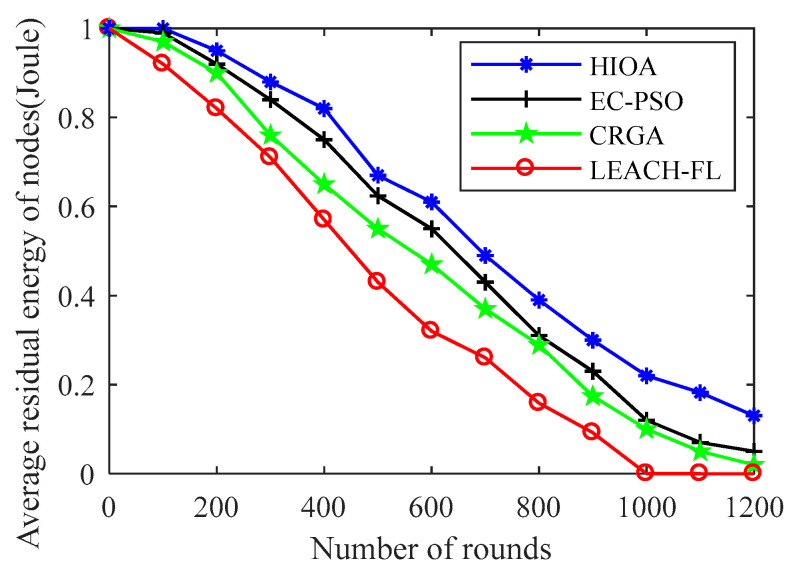
Average residual energy over rounds.

A comparison of the average residual energy per IoT node with respect to the round between the proposed algorithms and state-of-the-art algorithms is shown in Figure 8. The average residual energy is calculated as dividing the overall residual energy per round by the total number of IoT nodes. It is obvious that the residual energy of IoT nodes that uses the presented HIOA has more residual energy (0.12 Joule) after completion of 1200 rounds, whereas CRGA [29] and EC-PSO [28] algorithms consume more energy in data communication and IoT nodes have only 0.05 Joule and 0.02 Joule of energy art 1000 rounds. This is due to the fact that the proposed algorithm HIOA optimally selects the CHs at each round and balances the energy consumption load among all the nodes within a cluster. This can be attributed to the proper division of IoT nodes in rooster, hens and chicks, where the mother–child (hens & chicks) relationship is also balanced. In addition, crossover and mutation operators also help in enhancing the selection of optimum set of CHs. It is also worth noting that initially the LEACH-FL [27] algorithm consumes less energy and IoT nodes have a handsome amount of residual energy left out. However, in the increment of rounds, the residual energy of IoT nodes declined sharply and almost zero within 892 rounds. This is because LEACH-FL ignores the inter-cluster and intra-cluster communication costs in the selection of CHs that consume extra energy. Thus, overall, the proposed algorithm HIOA has more residual energy compared to EC-PSO, CRGA and LEACH-FL by 11%, 16% and 21%, respectively.

#### 4.2.5. Comparison of Standard Deviation over Rounds

**Figure 9:**
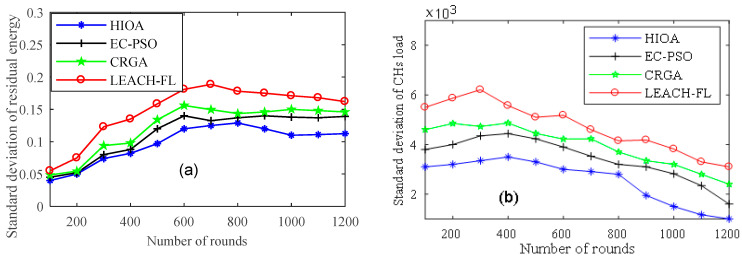
Standard deviation over rounds: (**a**) residual energy and (**b**) CH load.

A comparison of the standard deviation of residual energy and energy load balance by the CHs between the proposed algorithm and state-of-the-art algorithms is shown in Figure 9a and b, respectively. The standard deviation of residual energy measures the variability and consistency of the residual energy of the IoT nodes in the population. This is evident from the result that the standard deviation of residual energy and energy load of the selected CHs for the proposed HIOA algorithms is more balanced compared to EC-PSO [28], CRGA [29] and LEACH-FL [27]. This can be attributed to the reason that it’s minimizing the energy consumption during the selection of the fitness function of CHs by considering inter-cluster and intra-cluster distance. In addition, GA operators (crossover and mutation) help in reducing the convergence time in selecting the global optimum set of CHs, which increases population diversity and not to select local optimum CH sets. EC-PSO forms the clusters according to swarm particle optimization, and normal nodes join the cluster according to the distance from the edge node and left behind the number of neighboring IoT nodes. This can be attributed to unbalanced energy consumption in the cluster. 

It can also be noticed that CRGA and LEACH-FL show variations in the standard deviation of the IoT node’s residual energy and load balancing by CHs. It is due to the fact both CRGA and LEACH-FL algorithms choose the CHs randomly, in turn, unevenly distributing the load on the CHs and reducing residual energy. The worst performance is shown by LEACH-FL because probabilistic selection generates some isolated CHs that consume much more energy in the transmission of data to edge nodes. Thus, the overall result proved that the standard deviation of residual energy of the proposed algorithm HIOA was lower than the EC-PSO, CRGA and LEACH-FL by 18%, 26% and 42%, respectively. The load balancing of the proposed algorithm is also 23%, 32% and 48% lower than state-of-the-art algorithms.

## 5. Conclusions and Future Scope

In this paper, we proposed a new hybrid algorithm for clustering in the IoT network based on FL and CSO employing a GA to minimize energy dissipation. To this end, tentative CHs are selected using FL by considering essential parameters. Further, enhanced CSO with GA utilizes the concept of hierarchal order (rooster →hen →chicks) to divide the population and their fitness values are evaluated in order to find the best of nodes as CHs using crossover and mutation operation of GA. Simulations conclude that our proposed algorithm HIOA is robust to different CSO parameters and finds a near-optimal solution with low time complexity and higher convergence speed over state-of-the-art algorithms. The particle implementation of the simulate model can be used for load balancing or self-organizing structure in IoT nodes (electric vehicles) for the energy buying market.

The presented HIOA model effectively captures the rapid and frequently changing traffic patterns due to the inherent feature of GA and the dynamic classification property of CSO with a lower rate of latency for convergence. As the proposed model has no prediction ability for a sustainable network where electric vehicles are used as nodes, to add these features in the future, we included a neural network or deep learning approach for the formation of clusters based on past experience [34]. The nearby models include training and testing and deployment as well in different network scenarios.

## Figures and Tables

**Table 1:** Comparative study of recent research works.

	Characteristics	Issues	Techniques	Contributions	Metrics	Limitations	Publication Year
Protocols							
GAOC [21]		Selection of the optimum number of CHs	Genetic Algorithm	Multiple data sinks to overcome hotspot problem	residual energy, node density and node distance	Parameter intra-cluster distance have been not taken	2019
LEACH-FL [27]		Clustering process and routing	Fuzzy Logic Inference System	Improved clustering process	residual energy	Left out Node density	2020
EC-PSO [28]		Hotspot problem	Particle swarm intelligence	Improved fitness function	residual energy,	Left out node distance to base station causes exhaust more energy	2019
CRCGA [29]		Load-balance clustering process with routing	Genetic Algorithm	Select optimal CHs and best route	Encode them into single chromosome	Inter/Intra clustering distance ignored	2020
ICSO-LA [30]		Localization error	CSO	Prevent from IoT nodes to falling into local optimum	Update the distance from real node to base station	Node delay and node density is not covered that arises the problem of hotspot	2021
SEOANS [31]		Optimize beam pattern in WSNs	Cuckoo Search and CSO	Calculation method for node location	Adopt chaos theory and grade scheme to improve CSO	Node density is left out	2018
Interruptible load scheduling protocol [33]		Power balance between supply and demand	CSO	Solve the interruptible load scheduling on peak demand	Alleviate the peak load by reducing cost	Green energy resource and delay constraint is neglected	2021

**Table 2:** Fuzzy logic rules.

Rule	If			Then	Rule	If			Then
	*I_re_*	*I_nd_*	*I_de_*	*I_ch_*		*I_re_*	*I_nd_*	*I_de_*	*I_ch_*
1.	BW	SE	NB	BR	15.	UT	AE	FR	AR
2.	BW	SE	MD	BR	16.	UT	CT	NB	SG
3.	BW	SE	FR	NB	17.	UT	CT	MD	SG
4.	BW	AE	NB	NE	18.	UT	CT	FR	AR
5.	BW	AE	MD	BR	19.	GT	SE	NB	HD
6.	BW	AE	FR	BR	20.	GT	SE	MD	HD
7.	BW	CT	NB	NE	21.	GT	SE	FR	SG
8.	BW	CT	MD	NE	22.	GT	AE	NB	HT
9.	BW	CT	FR	BR	23.	GT	AE	MD	HR
10.	UT	SE	NB	NE	24.	GT	AE	FR	HD
11.	UT	SE	MD	AR	25.	GT	CT	NB	HT
12.	UT	SE	FR	NE	26.	GT	CT	MD	HR
13.	UT	AE	NB	SG	27.	GT	CT	FR	HD
14.	UT	AE	MD	AR					

**Table 3:** Simulation parameters.

Parameter	Value
Number of nodes (*N*)	200
Network size	150 × 150
Percentage of CH	5
Packet size	4000 bit with 100 bit header
Initial Energy	1 J
*E_ay_*	5 nJ/bit/message
*E_T_*	50 nJ/bit
$\partial_{fs}$	10 pJ/bit/m^2^
$\partial_{mp}$	0.0013 pJ/bit/m^4^
ϑ	0.3
*τ*	0.006
Cycle time	60 µs
Crossover rate	0.7
Mutation rate	0.1
Population size	60

## References

[1] Aanchal A., Kumar S., Kaiwartya O., Abdullah A.H. (2016). Green computing for wireless sensor networks: Optimization and Huffman coding approach. Peer-to-Peer Netw. Appl..

[2] Kumar V., Kumar S., AlShboul R., Aggarwal G., Kaiwartya O., Khasawneh A., Lloret J., Al-Khasawneh M. (2021). Grouping and Sponsoring Centric Green Coverage Model for Internet of Things. Sensors.

[3] Rehman A., Haseeb K., Saba T., Lloret J., Sendra S. (2021). An Optimization Model with Network Edges for Multimedia Sensors Using Artificial Intelligence of Things. Sensors.

[4] Rani R., Kumar S., Kaiwartya O., Khasawneh A., Lloret J., Al-Khasawneh M., Mahmoud M., Alarood A. (2021). Towards Green Computing Oriented Security: A Lightweight Postquantum Signature for IoE. Sensors.

[5] Kashyap P.K., Kumar S., Jaiswal A., Kaiwartya O., Kumar M., Dohare U., Gandomi A.H. (2022). DECENT: Deep Learning Enabled Green Computation for Edge centric 6G Networks. IEEE Trans. Netw. Serv. Manag..

[6] Al-Sodairi S., Ouni R. (2018). Reliable and energy-efficient multi-hop LEACH-based clustering protocol for wireless sensor networks. Sustain. Comput. Inform. Syst..

[7] Vijayalakshmi V., Senthilkumar A. (2019). USCDRP: Unequal secure cluster-based distributed routing protocol for wireless sensor networks. J. Supercomput..

[8] Al-Shalabi M., Anbar M., Wan T.-C., Alqattan Z. (2019). Energy efficient multi-hop path in wireless sensor networks using an enhanced genetic algorithm. Inf. Sci..

[9] Heinzelman W.R., Chandrakasan A.P., Balakrishnan H. Energyefficient communication protocol for wireless sensor networks. Proceedings of the 33rd Annual Hawaii International Conference on System Sciences.

[10] Zhu F., Wei J. (2019). An energy-efficient unequal clustering routing protocol for wireless sensor networks. Int. J. Distrib. Sens. Netw..

[11] Genta A., Lobiyal D.K., Abawajy J.H. (2019). Energy Efficient Multipath Routing Algorithm for Wireless Multimedia Sensor Network. Sensors.

[12] Kaiwartya O., Kumar S. (2015). Cache agent-based geocasting in VANETs. Int. J. Inf. Commun. Technol..

[13] Sert S.A., Alchihabi A., Yazici A. (2018). A two-tier distributed fuzzy logic based protocol for efficient data aggregation in multihop wireless sensor networks. IEEE Trans. Fuzzy Syst..

[14] Prasad M., Liu Y.T., Li D.L., Lin C.T., Shah R.R., Kaiwartya O.P. (2017). A new mechanism for data visualization with TSK-type preprocessed collaborative fuzzy rule based system. J. Artif. Intell. Soft Comput. Res..

[15] Cai X., Sun Y., Cui Z., Cui Z., Zhang W., Chen J. (2019). Optimal LEACH protocol with improved bat algorithm in wireless sensor networks. KSII Trans. Internet Inf. Syst..

[16] Sahoo B.M., Amgoth T., Pandey H.M. (2020). Particle swarm optimization based energy efficient clustering and sink mobility in heterogeneous wireless sensor network. Ad Hoc Netw..

[17] Deb S., Gao X.-Z., Tammi K., Kalita K., Mahanta P. (2020). Recent studies on chicken swarm optimization algorithm: A review (2014–2018). Artif. Intell. Rev..

[18] Sambo D.W., Yenke B., Förster A., Dayang P. (2019). Optimized clustering algorithms for large wireless sensor networks: A review. Sensors.

[19] Gong D., Yang Y., Pan Z. (2013). Energy-efficient clustering in lossy wireless sensor networks. J. Parallel Distrib. Comput..

[20] Sohrabi M.K., Alimirzaee S. (2019). Improving performance of node clustering in wireless sensor networks using meta-heuristic algorithms and a novel validity index. J. Supercomput..

[21] Verma S., Sood N., Sharma A.K. (2019). Genetic algorithm-based optimized CH selection for single and multiple data sinks in heterogeneous wireless sensor network. Appl. Soft Comput..

[22] Cui L., Zhang Y., Jiao Y. (2021). obust Array Beamforming via an Improved Chicken Swarm Optimization Approach. IEEE Access.

[23] Pitchaimanickam B., Murugaboopathi G. (2020). A hybrid firefly algorithm with particle swarm optimization for energy efficient optimal CH selection in wireless sensor networks. Neural Comput. Appl..

[24] Devassy D., Immanuel Johnraja J., Paulraj G.J.L. (2022). NBA: Novel bio-inspired algorithm for energy optimization in WSN for IoT applications. J. Supercomput..

[25] Shi W., Wang W., Yu Y., Zhang S., Cao Y., Yan S., Gao J. (2021). Optimal Deployment of Phased Array Antennas for RFID Network Planning Based on an Improved Chicken Swarm Optimization. IEEE Internet Things J..

[26] Nasri M., Helali A., Maaref H. (2021). Energy-efficient fuzzy logic-based cross-layer hierarchical routing protocol for wireless Internet-of-Things sensor networks. Int. J. Commun. Syst..

[27] el Alami H., Najid A. (2020). Fuzzy logic based clustering algorithm for wireless sensor networks. Sensor Technology: Concepts, Methodologies, Tools, and Applications.

[28] Wang J., Gao Y., Liu W., Sangaiah A., Kim H.-J. (2019). An improved routing schema with special clustering using PSO algorithm for heterogeneous wireless sensor network. Sensors.

[29] Wang C., Liu X., Hu H., Han Y., Yao M. (2020). Energy-Efficient and Load-Balanced Clustering Routing Protocol for Wireless Sensor Networks Using a Chaotic Genetic Algorithm. IEEE Access.

[30] Sandeli M., Bouanaka M.A., Kitouni I. An Efficient Localization Approach in Wireless Sensor Networks Using Chicken Swarm Optimization. Proceedings of the 2021 International Conference on Information Systems and Advanced Technologies (ICISAT).

[31] Sun G., Liu Y.H., Liang S., Chen Z.Y., Wang A.M., Ju Q.A., Zhang Y. (2018). A sidelobe and energy optimization array node selection algorithm for collaborative beamforming in wireless sensor networks. IEEE Access.

[32] Wang Q., Zhu L. (2017). Optimization of wireless sensor networks based on chicken swarm optimization algorithm. AIP Conf..

[33] Wang J., Zhang F., Liu H., Ding J., Gao C. (2021). Interruptible load scheduling model based on an improved chicken swarm optimization algorithm. CSEE J. Power Energy Syst..

[34] Kashyap P.K., Kumar S., Jaiswal A., Prasad M., Gandomi A.H. (2021). Towards Precision Agriculture: IoT-Enabled Intelligent Irrigation Systems Using Deep Learning Neural Network. IEEE Sens. J..

